# Diseases of the Upper Respiratory Passages and Their Relations to Oral Deformities*Read before Boston and Tufts Dental Alumni Association, Dec. 15, 1909.

**Published:** 1910-08-15

**Authors:** Wm. E. Chenery

**Affiliations:** Boston


### DISEASES OF THE UPPER RESPIRATORY PASSAGES AND THEIR RELATIONS TO ORAL DEFORMITIES.*

WM. E. CHENERY, M.D., BOSTON.

As a result of the recent almost world-wide activity in oral inspection, dentists and physicians realize as never before that the greater portion of the people of all ages are suffering for want of dental attention. Lack of knowledge of the value of the teeth and how to properly care for them, is the reason. The present agitation is timely and will result in great good.

With added knowledge comes greater responsibilities to both dentists and physicians and a need of closer affiliation. We are waking up to the necessity of a hygenic mouth with normal secretions, and the value of every tooth, each in normal occlusion as great factors in good health. The general physician has largely given over the care of the mouth to the dentist and the rhinologist and the responsibility is growing more important as facts develop. The opportunity is now given to the dentist of rising above the mere mechanical art of extracting, filling and supplying artificial teeth.

There is a chance to assert your right to a proper place as a specialist in a most important branch of medicine. With

*Read before Boston and Tufts Dental Alumni Association, Dec. 15, 190[illegible].

your intimate knowledge of the teeth and the care of the mouth preventive medicine may be materially advanced.

The tendency is to hurry through our anatomy, physiology, histology, pathology and general surgery but they are the essential basis for good work. You ought to have a better knowledge of the border line specialties which have their influence on the teeth and mouth. If pathological knowledge and general surgery technique and skill were developed by the dentists, the diseases of the mouth affecting the health of the individual might well be cared for by him. You are capable of caring for a part of these conditions, why not the whole?

It took a *singing master*, Manuel Garcia, to invent the forehead mirror and show its possibilities, and a *dentist* introduced anathesia to the surgical world. Why should not the dentist with his superior knowledge of the condition in the mouth and his instrumental and mechanical ability be skilled in the examination and treatment of the neighboring nose and pharynx which play such an important part in his own speciality.

We do not so much need a larger materia medica as we do a better knowledge of the normal in nature and how to maintain it. Pure air and pure food properly prepared are the greatest essentials. A robust constitution demands a certain amount of each to nourish the body and supply resistance to the inroads of disease. According as we limit the supply of pure air and pure food we restrict development and health. Definite amounts of proteids and carbohydrates are necessary to maintain the body weight and strength. But it does not so much matter what the food is, providing it be a mixed diet, as how prepared and how treated in the mouth. The sense of taste we know must be right to stimulate the flow of saliva. If the voluntary part of digestion (mastication) is right, it is rare to have digestive disturbances. In this connection the latest utterance of Horace Fletcher is worth quoting: "five years from now it will not be considered respectable to be sick." While this prophesy is overdrawn, we should nevertheless keep pace with the 1915 spirit of agitation, for *this*, at least, is true, if food either through negligence or mechanical inability is improperly prepared in the mouth, or if it is there made impure by pus from diseased gums, decayed food or bacteria from carious teeth, then stomach and intestinal diseases and impoverished blood will surely result. A little more care of the teeth and thorough mastication means less need of selective care in diet. The 20 or 30 square inches of surface in the mouth, with innumerable places of lodgment for bacteria, need to be kept clean all the time so that food shall not there be infected and rendered a menace to health. Irregularities of teeth afford more opportunities for decay, also imperfect occlusion interferes with the proper grinding and mixing of food. Often we bolt food, many times the cause of this bad habit is started by the teeth themselves. Irregularities of the teeth not only interfere with proper mastication but improper interdigitation of the cusps interferes with the normal symmetrical development of the jaws, and so affects the formation of the face. Thus the teeth play an important part in personal beauty.

The function of the saliva aside from its part in digestion is to moisten the mucous membrane and the food. It assists in forming the bolus which later is thrust back by the tongue to the fauces, and thence carried on to the esophagus. There remains much to be learned about this physiologic secretion; without it mastication is difficult and digestion imperfect. How does it differ in health and sickness? What are the results on the stomach? What effect have the different kinds of food on the saliva? What is its relation to caries of the teeth?

The mouth is nature's avenue for the food supply. Let us now see how nature supplies pure air to the lungs.

At the very entrance to the nose we find the moistened hair-like vibriss placed there to strain the air. The moisture on the mucous membrane further assists in collecting dust and bacteria and little cilia tend to whip the foreign material toward the entrance of the nose. We should expect to find numerous bacteria in the vestibules, and in fact they are there in so great numbers it is almost impossible even with the aid of a speculum to pass a sterile platinum wire loop into the interior nares without infecting it. However, the normal nose is sterile in its *inner* portion, due to the straining at the vestibules and the probable germicidal effect of the mucus.

Various attempts have been made to test the bacteriology of the interior of the nose. Some claim that staphylococci and streptococci are always present; others, according to their care in technique, have found occasionally sterile nares and few bacteria. The more careful the technique the less likely there are to be bacteria. Dr. F. C. Cobb* recently reported his findings in sixty cases; of these he found fifty-five were sterile while staphylococci were present in five. By sterilizing the vestibule with dioxygen of corrosive sublimate solution, even by taking his culture through a sterile rubber cot he found it hard to get an absolutely sterile nose, staphylococci usually being present. But by sterilizing the external nose and the vestibules with Harrington's solution for three minutes and using a speculum covered with a rubber cot he was able to get the vestibule free from all germs, and fifty-five out of sixty, sterile in the interior portion. He suggests faulty technique as the cause of failure in the other five cases.

HARRINGTON'S SOLUTION

Corrosive Sublimate ----- 2 grams
Hydrochloric Acid ------ 150 grams
Alcohol (70 per cent) ---- 2,500 grams

The nasal chambers are lined throughout with mucous membrane and on the outer wall of the nasal fosa are situated the turbinates. The air eddying through the nose comes in contact with these little coils and the mucous membrane, and so, becomes warmed or cooled, as is needed body heat, and at the same time the air is saturated with moisture. The lower turbinates, having erectile power, by

*Cobb, F. C. Transactions American Larynological Association, 1909.

swelling or contracting, make the nasal passages larger or smaller, thus acting as governors to the inlet of air.

If the nose is approximately normal and free from obstruction inspiration and expiration should on most occasions take place through the nose. It is nature's way. Then the air causes no irritation, but is properly purified and prepared for the delicate tissues of the larynx and lungs. Moisture in the normal nose is provided in just the exact amount needed so that there is neither dryness nor excess of secretion necessitating the use of the handkerchie for hawking and spitting.

If we breathe through the mouth for a considerable period, the mouth and pharynx becomes parched and inflamed. The throat burns and cough is produced from irritation and because of perverted secretion. A pharyngitis is produced and the general health is sure to be affected if long continued. The recent experiments of Dr. Willis S. Anderson* on the effects upon the respiratory and general systems by nasal obstruction are interesting. Guinea pigs, rabbits and dogs were used and closure of the nostrils was accomplished; with the smaller animals by means of cotton and collodion, with the larger ones by denuding the surfaces and suturing the nostrils. For prolonged experiments the latter method was found better. The guinea pigs that lived over forty-eight hours breathed partially through the nose; thirty-six hours of life was the average when the nostrils were closed tight. Distention of the abdomen occurred from air being swallowed; often this was very noticeable in an hour, especially if food were taken. The rabbits lived longer but the longest only 113 days, the shortest four days. Their abdomens did not distend, but their weight decreased and by the time half the body weight was lost they died, and autopsy showed dilations of the heart or some lung infection. Eighteen dogs were experimented on with partial or about two-thirds closure of the nostrils, and twenty-four puppies born of mothers who had nasal obstruction. Labored breathing, asthmatic in character, was a constant symptom, which increased on

*Anderson, W. S. Transactions American Laryngological, Physiological Ological Society, 1909.

exercise. It was noticed the hair become shorter, thiner and lighter in color and lost its natural gloss, and wrinkling of the skin appeared. The younger dogs died within three months of infection or broncho pneumonia. Of the puppies born of mothers whose nostrils had been partly closed, over half died before three months. They seemed handicapped and did not develope as normal dogs should, but gradually showed evidence of mal nutrition.

He draws these conclusions:

"Nasal obstruction leads to death or serious impairment of vitality."

"The lowered resistance predisposes to infections."

"Local diseases of the respiratory tract are induced."

"Obstruction of nostrils leads to dilatation of the heart."

"Changes in the skin and blood and symptoms of asthma and emphysema are induced." And lastly but very important, "reopening of the occluded nostrils is followed by prompt disappearance of the symptoms."

From these experiments it is evident that mouth breathing is most pernicious in its effect. It is as important in human beings as in animals.

What are the common conditions or diseases which interfere with normal nasal respiration? What are the results of interfering with this normal air course and its effects on physical and oral development? What can we do to prevent and when?

At birth the cranium is very large as compared with the face; this developes afterwards and its development is affected by various conditions which influence growth up to the twentieth year. Normal children usually start right by breathing as nature provides, through the nose; they seem to have no difficulty in so doing. I have said we should breathe equally through both nostrils. Anything interfering produces a pathological condition and the effects depend very much upon the length of time the obstrusction exists, and whether partial or complete. There is an atmospheric pressure of fifteen pounds to the square inch, any less than this causes a vacuum. If the child has a slightly deviated septum or the nostril is partially blocked, then a vacuum is produced back of the obstruction. The blood pressure within the tissues is the same, but there is less pressure from without or in the nasal fossa; this favors dilation. An increased blood supply means more secretion and finally growth of tissue, in other words hypertrophy, which at first depending on the cause, is transitory and then permanent or hyperplastic. This is especially noticeable in the inferior turbinates, and the anterior ends of the middle turbinates. As a rule the face is perfectly formed in utero, but by prolonged labor or by the face resting on the pelvic bones or by instrumental delivery, the septum may be slightly injured at birth. Again, as so often happens, the child developes a slight nasal cold in the early days. Trouble is started, for whatever blocks free nasal respiration interferes with the necessity of life—oxygen. It must be obtained, so mouth breathing is favored. At the very beginning of life a pernicious habit is encouraged, and if the obstruction becomes permanent there are tissue changes, more or less permanent and mouth breathing becomes a necessity. Every cold tends to make conditions worse. An acute rhinitis becomes a chronic rhinitis, the nasal chambers are no longer sterile, but full of mucopurulent secretions. With a vacuum in the nose and atmospheric pressure upward from inhalation of air through the mouth there is a tendency to raise the palatine arch. The roof of the mouth is the floor of the nose, so there is a tendency to narrowing of the nostrils, at least the developmental widening is handicapped. With interference of nasal breathing the lymphoid tissue in the postnasal space swells, becomes hypertrophied and thus gradually we have produced the adenoids. The acute exanthematous diseases of childhood, such as scarlet fever and measles, seem to specially favor lymphoid enlargement. Each succeeding cold causes the adenoids to enlarge and at length become more fibrous. The result is marked interference with nasal breathing and the necessity of mouth breathing much of the time, especially when lying down.

During the early days the nares are small and only slight obstructions are necessary to stimulate mouth breathing. Over-heated rooms, too many clothes and improper feeding all have bad influences. They make the child unable to withstand sudden changes in temperature and bacteria invasion. Lack of nasal cleanliness in young children is often a strong factor in producing mouth breathing. The natural protection of the nose is lost, and pharyngeal irritation, inflammation and infection takes place. The tonsils which are only a part of the same lymphoid tissue in the back of the throat become hypertrophied and infected. Of course we must always consider the shape of the face and nose inherited. But I believe mouth breathing early established is a very strong factor in moulding the round baby face into one long and pinched, with narrow nostrils. A seemingly slight evil habit early, begun is of great importance in shaping the jaws during the plastic stages of detention. Non-use of the alae muscles which dilate the nostrils allows collapse, non-development later impedes and makes difficult nasal respiration at the very commencement of the air tract.

What are the diseases that produce mouth breathing in children under the age of fifteen? The age of puberty is taken because by this time the children's diseases are over and the teeth are nearly erupted. Habits and deformities are well formed by this time. In the throat department of the Boston Dispensary not long ago I looked up the records of 3,000 cases of children under fifteen. The chief conditions for which they applied for help were acute rhinitis 3¾%; chronic rhinitis 5%, deviated septum 4½%, acute tonsillitis 6%, and 64.8% for adenoids or enlarged tonsils. These comprised about 85% of all cases treated of this age or under These figures tell this story: A so-called slight cold or rhinitis produces inflammation of the nasal mucous membrane of the upper air tract weaker and more susceptible to succeeding colds. A deviated septum favors irritations and lodgment of secretions. The acute condition leads to a sub-acute or chronic state with increased secretion and bacteria. Hypertrophy is the rule, nasal respiration is interfered with, and lymphoid enlargement results. The variety of diseases in the upper respiratory tract of children is not very large, but because they are more or less obstructive and accompanied with tissue changes they all tend to produce mouth breathing.

Why is mouth breathing so injurious especially in childhood? First, air, improperly prepared, is carried to the lungs Oxygenation does not take place as it should Respiration through the mouth is not as deep as through the nose, therefore interchange of gases in the lungs is not sufficient. The injurious carbon dioxide is not liberated from the lungs and the blood does not get as much oxygen as is needed. It has been proven there is a rapid increase in the red corpuscles after removal of nasal obstructions. From lack of normal air pressure in the nose and nasal pharynx and irritation, hypertrophy is favored. Increased blood supply means excessive nutrition and growth, then further blocking and opportunity for bacterial cultivation and invasion. This affects especially all of the lymphoid tissues in the back of the throat which are included in Waldeyer' ring, the pharyngeal tonsil or adenoids, the faucial tonsils and a fourth tonsil situated at the base of the tongue. Adenoids constantly secrete and besides interfering with voice sounds, make nasal respiration more or less difficult. The proximity of the adenoids to the eustachian tubes is a constant manace to the ears for middle-ear infection and deafness frequently result. At first in mouth breathing the jaw drops a little and the lips slightly part, after a time the mouth opens a little wider, the upper lip shortens and becomes thicker and later because the upper lip is not used and the nostrils are not dilated there is collapse of the alae and lack of developmental widening of the nares. Later a pinched appearance to the face is produced, imparting a stupid look. The mental development is handicapped. Frequent nasal colds are common, restless sleep which is most exhausting and debilitating is the rule. The effect on the formation of the dental arches is interesting. With the eruption of the teeth the face grows. Usually the deciduous teeth erupt with perfect occlusion but we should be sure the first and second temporary molars are in right position, for *they* may be factors in early mouth breathing because their little cusps do not interlock properly. The lack of opposing push from the tongue, because it lies in the floor of the mouth, and not against the teeth, may cause a tilting of these teeth. By the strong buccinator muscles we should find one little cusp of the lower molar in advance of the upper molar, and the upper molars should rest astride of the outer or buccal row of cusps of the lower teeth; if not we have an irregularity. If these teeth are not right then the first permanent molars, which should be guided into proper position by them, will go wrong. If there has been one sided, anterior nasal odstruction or loss of teeth on one side the arches may not be symmetrical. We must remember the temporary teeth are replaced by much larger teeth, so at about three and a half to the fifth year there are signs of enlargement of the dental arches, at this time the temporary incisors begin to separate from each other and at about six the first permanent molars, which are the real keystone to further development, appear. We must not forget the value of closed lips in moulding the anterior arches. The closed lips should counter balance the thrust of the top of the tongue and thus assist the incisors into their proper place. In the early years of life, sickness such as measles, scarlet fever, rickets, producing mal-nutrition, may leave its marks on the permanent teeth, but the result of nasal or postnasal stoppage is mouth breathing, which produces lateral crowding of the teeth leading to marked irregularities. If the chief trouble is enlarged tonsils we are apt to find a protruding lower jaw; while if adenoids are most prominent we have the V shaped arch, with certain teeth turned toward each other often overlapping, and a decided over bite. This is a very common condition.

If the nasal, postnasal or faucial obstructions are removed *early* and the mouth breathing habit overcome, so that the air travels in the right way and the lips are closed not too tightly, normal occlusion will usually result. The lips, cheeks and tongue with mouth closed will assist in moulding the arches and direct the teeth during the expansion period to make room for the larger, and also the extra teeth.

Because obstructions in the upper air passages are so frequent their signs should be watched for by physicians and dentists. Frequent colds are not simple things! The anterior nares must be kept clear; if turbinates enlarge and do not yield to soothing local treatment and constitutional building, they must be reduced by surgical means to the normal and deviations must be corrected, otherwise more marked and difficult conditions arise. If there is not a normal air current through the nares it will not broaden as it should, the floor of the nose will arch upward. From this narrowing we get difficult and insufficient inspiration and inability to thoroughly clean the nares of secretion.

We must not consider as trifling mouth breathing in early childhood. It must be remedied as soon as possible, it is usually easy at first; it may be possible later in life. If nasal breathing is unobstructed and care is taken to see that the child does breathe good air and properly, there will be fewer adenoids. If they are present they should be removed before bad habits are fixed. The best time I believe is from the third to fifth year, for it is necessary that obstruction to nasal inspiration be removed and the habit of nasal breathing by night as well as day be established before the changes resulting from second dentition take place. It is at this critical period that attention on the part of dentists and rhinologists is needed, for then a little prevention will mean much for the future health of the individual. Symmetrical enlargement of the jaws and nasal fossa are interdependent and their development depends much upon unimpeded respiration through the nose with mouth closed. If proper nasal breathing is carried on, the normally healthy child will develope nose, jaw and teeth right. Of course the thumb sucking and other bad habits must be elinimated. It is impotrant to retain every deciduous tooth as long as it is needed to guide into place its successor. Every tooth has a function and should be cared for and saved. A careless extraction will change the whole chain of events and interfere not only with the proper development of the dental arches, but be reflected in the developmental widening of the nasal fossa.

In conclusion,—the first need of the new born babe is oxygen. If sufficient air is supplied by day and night in the proper channel provided for it (the nose) and the stomach is supplied with a sufficient amount of food, the child will grow rugged and strong, with teeth in good occlusion. With improper breathing oxygenation is imperfect and the general health and development is much handicapped.

Mouth breathing during the peiod of dentition is very harmful, especially during the early part of second dentition. The balance of forces is upset by mouth breathing and at this crucial time symmetrical development of the nares, maxillary and dental arches is interfered with. Nasal and postnasal obstruction should be removed before this time and habits corrected in order to restore this balance of forces and permit normal development.

It is important to see to it that irregularities of teeth are early corrected whether of first or second dentition and preservation of all the permanent teeth should be maintained. Unilateral breathing is reflected in the dental arch development on the same side and also unilateral loss of teeth will influence the develpoment of the nasal fossa. The time to restore nasal breathing is early, before serious damage is done. Complete removal of adenoids by curette and tonsillectomy by blunt dissection and snare are the approved modern methods.

Finally: Right development depends on air and food properly prepared, sufficient in quantity and quality and the maintenance of nature's plan, also early correction of little faults which uncorrected later dwarf the development of the whole. Each part depends on the other.—*The Journal.*

